# Can Examination of WWW Usage Statistics and other Indirect Quality Indicators Help to Distinguish the Relative Quality of Medical websites?

**DOI:** 10.2196/jmir.1.1.e1

**Published:** 1999-08-11

**Authors:** Angel A Hernández-Borges, Pablo Macías-Cervi, María Asunción Gaspar-Guardado, María Luisa Torres-Álvarez DeArcaya, Ana Ruiz-Rabaza, Alejandro Jiménez-Sosa

**Affiliations:** ^1^Pediatric Intensive Care UnitHospital Universitario de CanariasSpain; ^2^Family and Community Medicine UnitHospital Universitario de CanariasSpain; ^3^Research UnitHospital Universitario de CanariasSpain; ^4^Primary Care Center of La OrotavaHospital Nuestra Señora de La CandelariaTenerifeCanary IslandsSpain; ^5^Department of Clinical ChemistryHospital Nuestra Señora de La CandelariaTenerifeCanary IslandsSpain

**Keywords:** Health Education, Information Systems, Computer Communication Networks, Internet, Bibliometrics, Cybermetrics, Web metrics, Webometrics

## Abstract

**Background:**

The Internet offers a great amount of health related websites, but concern has been raised about their reliability. Several subjective evaluation criteria and websites rating systems have been proposed as a help for the Internet users to distinguish among web resources with different quality, but their efficacy has not been proven.

**Objective:**

To evaluate the agreement of a subset of Internet rating systems editorial boards regarding their evaluations of a sample of pediatric websites. To evaluate certain websites characteristics as possible quality indicators for pediatric websites.

**Methods:**

Comparative survey of the results of systematic evaluations of the contents and formal aspects of a sample of pediatric websites, with the number of daily visits to those websites, the time since their last update, the impact factor of their authors or editors, and the number of websites linked to them.

**Results:**

363 websites were compiled from eight rating systems. Only 25 were indexed and evaluated by at least two rating systems. This subset included more updated and more linked websites. There was no correlation among the results of the evaluation of these 25 websites by the rating systems. The number of inbound links to the websites significantly correlated with their updating frequency (p<.001), with the number of daily visits (p=.005), and with the results of their evaluation by the largest rating system, HealthAtoZ (p<.001). The websites updating frequency also significantly correlated with the results of the websites evaluation by HealthAtoZ, both about their contents (p=.001) and their total values (p<.05). The number of daily visits significantly correlated (p<.05) with the results of the evaluations by Medical Matrix.

**Conclusions:**

Some websites characteristics as the number of daily visits, their updating frequency and, overall, the number of websites linked to them, correlate with their evaluation by some of the largest rating systems on the Internet, what means that certain indexes obtained from the usage analysis of pediatric websites could be used as quality indicators. On the other hand, the citation analysis on the Web by the quantification of inbound links to medical websites could be an objective and feasible tool in rating great amounts of websites.

## Introduction

After the early enthusiasm generated by the potential use of the Internet in Medicine [1,2,3], concern has been raised about the quality of the resources available on the Internet compared to more academic media. It is technically very easy to publish on the Internet [4]. The lack of a review process of the documents on the Net, and the power of this media in transmitting the data has the risk of misinforming both lay people [5,6,7] and health care professionals [8]. However, only a few studies have tried to measure this risk of misinformation [9,10,11]. Nothing yet is known about the users' ability to discriminate between low and high quality resources.

Several initiatives have been proposed which could be applied at different levels to improve the average quality of medical websites. For instance, we could apply certain basic methods for the websites to be correctly designed. In this sense, some academic organizations have proposed a set of basic information that every medical web site should provide about the author and sources of the web site contents, their potential conflicts of interest and funding, and the currency of the information [12]. But many of the available medical websites have been created without any quality control by a third party. How can Internet health care visitors distinguish between such different resources?

Internet users can find health and medical related websites in several ways. World Wide Web search engines (e.g., AltaVista, Excite, Infoseek and many others) provide the users with a list of websites that match a given topic, with the results ordered by syntactic similarity with the query [13]. Unfortunately, the quality of contents is not guaranteed.

On the other hand, certain websites indexes and review services, such as Medical Matrix (http://www.medmatrix.org/) and HealthAtoZ (http://www.HealthAtoZ.com/), offer systematic evaluations of medical resources on the Web [14], as a post publication editorial process. These rating systems could be an useful tool for guiding the visitors of medical websites [12]. However, authors who have reviewed these Internet resources, point out the variability of their evaluation criteria and their doubtful efficacy [14].

The quality of a given medical article on the Internet could be measured by the users opinion about it, for example by counting the number of times it is retrieved [15]. However, this idea has been criticized because it would replace the scientific peer review process with the opinion of the Internet users, whatever their qualification [6].

Despite the differences between the printed medical information and the Internet, several evaluation tools from the former could be useful if applied on the "Net." Similarly to printed medical journals, medical documents on the Internet could be ranked by their citation analysis [15,16], but no methods have been proven for use with medical websites. When an article is quoted in a paper, certain agreement among the authors may be supposed. Similarly, when a webmaster makes a link from his web site to another, certain credibility is given to the latter. In fact, the International Committee of Medical Journals Editors recommend caution when a link is made from a peer reviewed journal site to other sites [17]. If linking on the web can be equivalent to quoting in printed medical articles, a citation analysis on the web could be performed by the quantification of the links to a given medical web site.

The ideal method for assessing the quality of medical websites should provide a means of rating great amounts of medical web resources while respecting the World Wide Web peculiarities, such as its multimedia capabilities and changing contents. At the same time, it should at least be as reliable as systematic reviews of those resources by editorial boards. In summary, it should be a method born in the Internet but with the efficacy of those used in the printed media.

In this study, we evaluated the reliability of four websites characteristics as medical websites quality indicators. The four characteristis used: their authors' impact factor, their grade of updating, their daily visits and inbound links. The evaluations of a sample of pediatric websites by a number of Internet rating systems was the gold standard with which these websites characteristics were compared.

## Methods

During March 1998 a subset of websites rating systems were compiled. From these, we selected a sample of websites that were studied during the first week of April 1998.

Eight web rating systems, whose evaluations were offered as figures, were compiled from previous studies [13,14] (Table 1). One half of the selected rating systems gave the results of their evaluations by means of graphic analog scales, and the other half by numeric scales. Every web site evaluated by these rating systems that provided information about child health, whether for lay people or health professionals, was included in the study. Some of these rating systems (e.g., Lycos Top 5%) provides the visitors with a search tool by keyword. In these cases, the websites were selected using the keywords "Pediatrics", "Infancy", "Child health", and "Child Care." For the remaining rating systems, the pediatric websites were compiled manually. Those websites not accessible twice during the study period were excluded.

Only three rating systems (Medical Matrix, Physician Choice, and Six Senses) gave information about their editorial boards. Most of their members were physicians. Two of the web rating systems only gave a global result of their websites evaluation (Medical Matrix and Magellan), while the rest (HealthAtoZ, Argus Clearinghouse, Lycos Top 5%, Sympatico Health, Physician Choice, and Six Senses) gave a result for each considered criterion. Content was a common criterion to all the eight ranking systems. Therefore, the results of the evaluation of each web site were divided in two categories, content and non-content (design) aspects. In order to make comparisons, the results of the evaluations of the websites supplied by each rating system were transformed to a one hundred scale.

**Table 1:** Compiled web sites ranking systems. The results of evaluations are showed as two possible types of scales, graphic analog (A) or numeric (N)

**Rating systems (Included/excluded web sites)**	**Uniform Resource Locator**	**Type of scale**
Argus Clearinghouse Seal of Approval (16/1)	http://www.clearinghouse.net/cgi-bin/chadmin/viewcat/Health___Medicine?kywd++	A
HealthAtoZ (241/66)	http://www.healthatoz.com	*
Lycos Top 5% (8/3)	http://point.lycos.com/topics/Health_Overall.html	N
Magellan Internet Guide (40/11)	http://www.mckinley.com/magellan/Reviews/Health_and_Medicine/index.magellan.html	A
Medical Matrix (75/11)	http://www.medmatrix.org/SPages/Pediatrics.asp	A
Physician's choice (4/0)	http://www.mdchoice.com/pcsites.htm	N
Six Senses Seal of Approval (4/0)	http://www.sixsenses.com/winners.html	N
Sympatico Health (8/1)	http://www1.sympatico.ca/Contents/Health/LISTS/D3-C03_all1.html	A

^*^ Graphic analog scale developed in numeric

When provided, the daily visits registered by the websites visits counters were recorded. In some websites the date from which the counter was started was not available. Thus, their webmasters were asked for this information by electronic mail, and it was included in the statistical study if provided before the end of the observation period, 15th April 1998.

The websites authors and editors' names were searched in 1997 MEDLINE [18], and their articles were registered. Their impact factors of the journals wherein they were published were obtained by using the 1996 Science Citation Index (Institute for Scientific Information, Philadelphia, PA). The impact factor of a given web site author was the sum of the impact factors of his or her articles. For institutional websites only the name of the web editor was considered.

When provided, the time since the last update was also recorded.

Finally, by means of the Web search engine Infoseek [19], we calculated how many websites on the Internet linked to each web site of our sample. The searching strategy by syntax of this engine allows to know the websites that are linked to a given web site [20]. As a web site may be linked not only from external websites but also from websites of its own organization, we only considered external links. Although other search engines such as AltaVista, Excite or HotBot offer similar searching options, we chose Infoseek because it provided the results of the queries grouped by web site, which makes the exclusion of the internal links easier.

Comparison of means was performed by Mann-Whitney U test, and correlation analysis by means of Spearman's correlation coefficient ( *r_S_*). P values equal or less than .05 were considered significant. All computations were made with SPSS for Windows 7.0 (SPSS Inc., Chicago, IL) statistical package.

## Results

After excluding 93 non-accessible websites, a total of 363 pediatric websites were compiled.

**Table 2:** Correlations among the number of daily visits to the web sites, the impact factor of their authors or editors, the grade of update, and the number of links that receive. NS means not significant

	**Number of inbound links *r_S_* p**	**Visits/day *r_S_* p**	**Author's impact factor *r_S_* p**
Visits/day	.46 .005		
Author impact factor	NS	NS	
Weeks since the last update	-.36 <.001	NS	NS

**Table 3:** Correlation among the number of links and visits to the web sites, the impact factor of their authors, and the time since the last update, and the results of their evaluation by HealthAtoZ and Medical Matrix. No significant correlations were demonstrated with the other systems. Medical Matrix only provides total results, does not specify results by contents and non-contents aspects

		**Number of Inbound Links**		**Visits/day**		**Author impact factor**		**Weeks since the last update**	
		***r_S_***	**p**	***r_S_***	**p**	***r_S_***	**p**	***r_S_***	**p**
HealthAtoZ	Total Contents Non contents	.29 <.001 .30 <.001 .24 <.001		NS		NS		-.19 .04 -.23 .00 NS	
Medical Matrix	Total	NS		.79 .03		NS		NS	

On average, the websites of our sample received links from 470 other sites on the Internet (range, 0 to 3574). In 48% of the websites, information on their last update was given. On average, they had been updated 47.5 weeks before (range, 0 to 395). Only 10% of the websites had a visit counter, and the average daily visits were 470 (range, 1.2 to 3145). Seven visit counters did not distinguish among different visitors, that is, they registered any visit to their websites. In 137 websites (38%) the editor/author's name was given, but only 60 of them had published at least one article since January 1997 in the journals included in MEDLINE database. Their average impact factor was 2.14.

**Figure 1:**
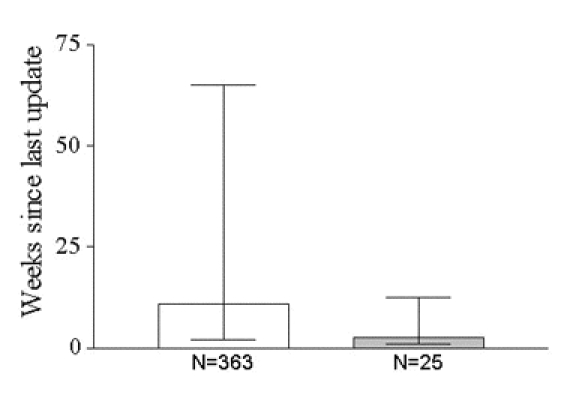
Weeks since the last update for the total of the sample, n=363, and for the websites evaluated at least by two rating systems, n=25 (median, 25^th^ and 75^th^ percentiles)

**Figure 2:**
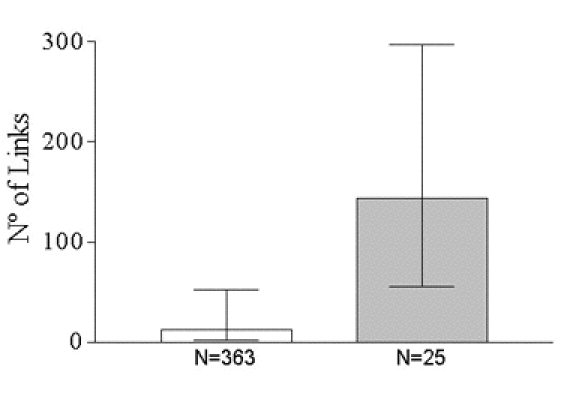
Number of inbound links to websites for the total of the sample, n=363, and for the websites evaluated at least by two rating systems, n=25 (median, 25^th^ and 75^th^ percentiles)

**Table 4:** Top 50 pediatric web sites of the sample (N= 363) by the number of their inbound links. The weeks since the last update, the number of daily visits to the web sites and their editor/author's impact factor are also provided. In parenthesis, the place that each web site would obtain if ranked by the two latter criteria. In italics, those web sites indexed at least by two rating systems. Missing values are due to the lack of visits counter, editor's name, or information about the last update, for many web sites

	**Uniform Resource Locator**	**Number of inbound links**	**Daily visits to web sites**	**Web site editor/author's impact factor**	**Weeks since the last update**
1	http://www.merck.com	3574	-	-	13
2	http://www.ucalgary.ca/~dkbrown/index.html	2355	1620 (3º)	0 ( ^3^60º)	-
3	***http://KidsHealth.org***	1109	-	-	-
4	***http://www.psych.med.umich.edu/web/aacap***	927	-	-	3
5	***http://www.aap.org***	896	-	-	1
6	http://www.chadd.org	785	-	-	4
7	***http://www.castleweb.com/diabetes***	767	-	-	-
8	http://www.medconnect.com	714	-	-	-
9	http://www.aaaai.org	677	-	-	-
10	http://www.aacap.org/web/aacap	612	-	-	4
11	http://www.nas.com/downsyn	572	-	0 ( ^3^60º)	1
12	http://www.childbirth.org	534	-	-	-
13	http://web.syr.edu/~jmwobus/autism	502	-	0 ( ^3^60º)	-
14	http://oncolink.upenn.edu/disease	487	-	10.1 (8º)	9
15	***http://www.jdfcure.com/index.html***	428	-	-	-
16	http://www.mic.ki.se/Diseases/index.html	423	1412 (5º)	-	-
17	http://www.asf.org	365	940 (6º)	-	1
18	http://www.mdcc.com	365	-	-	1
19	http://www.mc.vanderbilt.edu/peds	357	-	-	-
20	http://www.ama-assn.org/journals/standing/jama/jamahome.htm	330	-	-	-
21	***http://www.med.jhu.edu/peds/neonatology/poi.html***	322	253 (10º)	9.3 (13º)	2
22	http://www.wish.org	317	-	-	-
23	http://education.indiana.edu/cas/adol/adol.html	312	-	0 ( ^3^60º)	52
24	***http://www.kidsdoctor.com***	297	-	0 ( ^3^60º)	-
25	http://www.xmission.com/~gastown/safe	297	94 (20º)	-	-
26	http://www.childquest.org	287	-	-	6
27	***http://www.uab.edu/pedinfo***	284	-	-	-
28	http://www.childsecure.com	255	-	-	-
29	http://www.mc.vanderbilt.edu/peds/pidl	254	-	0 ( ^3^60º)	1
30	http://www.stjude.org	251	-	-	-
31	***http://www.nccf.org***	249	70 (25º)	-	8
32	***http://www.mda.org.au***	238	-	0 ( ^3^60º)	12
33	http://www.peds.umn.edu	235	-	0 ( ^3^60º)	2
34	http://www.csmc.edu/neonatology	232	117 (15º)	-	1
35	http://med-aapos.bu.edu	225	-	0.4 (51º)	3
36	http://www.jhbmc.jhu.edu	220	-	-	3
37	http://sids-network.org	214	3145 (1º)	0.3 (54º)	1
38	http://www.diabetes.com	212	-	-	-
39	http://sids-network.org/index.htm	208	3145 (1º)	0.3 (55º)	1
40	http://www.oneworld.org/scf	205	-	-	-
41	http://www.childmmc.edu	204	-	-	13
42	http://www.os.dhhs.gov/hrsa/mchb	197	-	-	7
43	***http://www.wp.com/pedsrheum***	197	81 (23º)	11.7 (6º)	-
44	http://dem0nmac.mgh.harvard.edu/neurowebforum/neurowebforum.html	188	2441 (2º)	0 ( ^3^60º)	-
45	***http://pedsccm.wustl.edu***	179	145 (13º)	1.0 (39º)	2
46	http://www.drgreene.com	179	-	-	-
47	http://www.medsch.wisc.edu	162	-	-	-
48	http://www.blindcntr.org/bcc	150	-	-	-
49	***http://home.coqui.net/titolugo***	144	68 (26º)	0.2 (56º)	1
50	http://www.chmcc.org	141	-	-	1

Only 25 websites of the sample were indexed and evaluated at least by two rating systems, and none by the eight. This subset of websites showed significantly better results of the evaluation of their contents and design by HealthAtoZ, and higher grade of updating (Figure 1) and higher number of inbound links (Figure 2). When the evaluations of these 25 websites by the different rating systems were compared, no significant correlations were found. Changes regarding the average impact factor of the authors of the websites or the number of daily visits could not be demonstrated in this subset of websites.

Some interesting correlations between the results of the evaluations of the websites and the rest of study variables were found. The number of links received by the websites significantly correlated with their daily visits and with the time since the last update (Table 2). The number of inbound links also correlated with the results of the websites evaluation by HealthAtoZ (Table 3).

The number of daily visits significantly correlated with the results of the websites evaluation made by Medical Matrix, and the grade of updating significantly correlated with the results of the contents and designs evaluation made by HealthAtoZ (Table 3).

Finally, no correlation was demonstrated between the average impact factor of the websites authors and the other variables.

The top fifty pediatric websites of the sample are shown in Table 4, ordered by the number of their inbound links according to the Infoseek indexing engine. More than a half of the 25 websites indexed by at least two rating systems may be found among these top fifty websites.

## Discussion

In this study, certain websites characteristics that depend on the users' preferences have been compared with evaluations of pediatric resources on the Web by third parties. Although rating systems have been previously criticized because their editorial boards frequently do not employ uniform criteria [13], we have considered them as the standard method because it somewhat represents a post-publication review process.

Some aspects of our method are open to discussion. Firstly, the reliability of the data regarding the daily visits and the updating frequency depends on the accuracy of the information that the websites editors offer in their sites. In this sense, we considered the grade of updating of the websites by the dates of their last changes. Clearly these changes could involve very different aspects and in different grades, and not necessarily provide more current contents. However, we believe that it could demonstrate the editor's efforts in maintaining or increasing the interest of his web site for the visitors.

The results regarding the number of daily visits to the websites must be considered with caution when comparing one web site to another, because some visit counters were set to register every visit, instead of every distinct visitor. Nevertheless, both can be considered usage indexes of a given web site.

On the other hand, quantification of links to the websites clearly depends on the power of the search engine we employ. By no means our results show the *total* number of links to the websites in our sample. In fact, a previous article states that it would be necessary to combine the databases from at least five large search engines to cover the most of the web [21].

Although all bibliometric indexes have limitations [22,23], we employed the impact factor as a measure of the webmasters' publishing capacity because it is a classical indicator of the quality of biomedical articles. Recently, it has been suggested that every medical web site should be evaluated following some basic criteria [24]. One of the more accepted criteria is that the authorship must be clearly stated, as a basic means for assessing the reliability of the web site contents. However, we could not demonstrate that the more highly evaluated, the most updated, or the most linked or visited pediatric websites, had the authors with the highest publishing capacity measured by their impact factor. In other words, some web quality standards do not correlate with classical quality standards from the printed media such as the impact factor of a given author's articles.

We could not find statically significant correlations among the evaluations of the websites by the different rating systems. This is probably due to the small size of the subset of websites indexed and evaluated by all the systems, and their different evaluation criteria. However, some interesting data were found when we considered the correlations among the four websites characteristics and the evaluations. We found that the best websites for HealthAtoZ, the largest analyzed rating system, were the most updated and the most linked ones. On the other hand, the most valuable websites for Medical Matrix, the second rating system by size, were the most visited ones. In any case, both the number of daily visits and the time since the last update highly correlated with the number of inbound links. The lack of correlation among the four variables and the evaluations by the other rating systems could be due to their little contribution to our sample.

Many efforts to establish quality criteria will have limited efficacy due to the dynamic behaviour of the Internet as a publishing medium. In fact, a recent article demonstrates the lack of consensus among the editorial boards of a large sample of evaluation and rating systems regarding the evaluation criteria they employ. The same authors pointed out that "... it may be difficult or even inappropriate to develop a static tool or system for assessing health related websites." [25] Therefore, the question could be to provide context to this issue. That is, to know how good a given medical web site is in comparison with the rest of medical websites. A democratic and feasible method for reaching this objective could be let the Internet community say which medical websites are the best ones, that is, which they usually visit or which they usually recommend by linking to them. Moreover, we believe that the fact that these usage indexes correlate with the evaluations by third parties, qualifies them as quality markers.

Eysenbach and Diepgen [16] have recently proposed that an ideal quality control system for medical resources on the Internet should take in account the users opinion, and not only their evaluation by a third party, that is, a "downstream filtering" and not only an "upstream filtering" approach. More interestingly, our study demonstrates certain agreement among both approaches in identifying high quality resources.

LaPorte et al [15] proposed an electronic publishing system in which the impact of a given resource on the Internet could be measured by counting how many times the document was retrieved or quoted. The introduction of the citation analysis of the medical resources on the web as a method to assess their quality has been recently proposed [16]. On the other hand, a very promising software system is being developed by Kleinberg [26,27]. This system would provide the users with a way of knowing the very best of the web on a given topic in a faster and more complete way than commercial human compiled directories. This system is based in the identification of two subsets of websites when a query on a given topic is made, those websites containing a lot of information about the topic (*authoritative websites*) and those which contain large amounts of links to the former (*hub sites*). Our work demonstrates that those authoritative websites, that is the more linked ones, are indeed the best ones regarding the evaluation of its contents and design by the editorial boards of some large web rating systems.

The citation analysis of biomedical journals has been a classic tool in assessing their relative quality [28]. Similarly, medical web resources could be ranked by a "webcite index" [16], which is not yet defined. Linking in the World Wide Web could be equivalent to quoting in printed publications, and its quantification could be useful for measuring the relative quality of medical websites. Some indexes could be created to make more rational comparisons among websites with different sizes. For example, in the same way that the calculation of the impact factor of a given medical journal takes into account the number of articles published by that journal yearly, the size of a given domain could be considered to obtain some indexes that would express more accurately the grade of linkage of a medical web site. Moreover, Platform for Internet Content Selection (PICS) [29], an infrastructure that could be applied as a filtering system of the medical information on the Net [16], could incorporate these indexes as one of the meta- data assigned to every medical document as electronic labels. Then, these electronic labels could be checked automatically by an user's browser, bypassing those documents with a "webcite index" not high enough. A problem could be how to avoid false "self-labelling" by dishonest webmasters. In any case, more work is needed to give answers to these and other technical questions on the emerging field of *Webometrics* [30].

An evaluation system based on this quantification would bring advantages and risks. Rankings could be generated very quickly and in an objective way, because the Internet community by itself would evaluate great amounts of medical websites. However, this evaluation process would be made *a posteriori*, and the potential harmful effects of the diffusion of documents without enough quality could not be avoided. Therefore, this method could not replace previous editorial effort that warrants a minimal quality for each resource.

Our work demonstrates that the visitors of pediatric websites and the editors of websites on the "Net," so called webmasters, show certain maturity when they have to identify the pediatric resources with high quality. We believe that the key point is how to augment the proportion of these resources. An important issue could be to establish a citation style not only for articles from peer reviewed electronic journals [31], but also for any medical document on the Net. The prestige that citation in a printed journal represents will stimulate high quality publishing on the Internet, and web site editors will employ enough review processes to obtain the necessary quality. A web site's ranking system based on the citation analysis on the web by the quantification of links would be an additional incentive. The more valuable resources will attract the Internet users' visits and the webmasters' links, and very likely the best funding and financial supports.

In summary, although the Internet provides a very different publishing medium, traditional means borrowed from printed journals could also be used with this electronic media for achieving minimal levels of quality. These include certain peer review processes, that enhance the rigor of the documents submitted for publication taking in account the peculiarities of this media, and linking analysis as a measure of the citation on the World Wide Web.
